# Intelligent grading method for walnut kernels based on deep learning and physiological indicators

**DOI:** 10.3389/fnut.2022.1075781

**Published:** 2023-01-05

**Authors:** Siwei Chen, Dan Dai, Jian Zheng, Haoyu Kang, Dongdong Wang, Xinyu Zheng, Xiaobo Gu, Jiali Mo, Zhuohui Luo

**Affiliations:** ^1^School of Mathematics and Computer Science, Zhejiang Agriculture and Forestry University, Hangzhou, China; ^2^Zhejiang Key Laboratory of Forestry Intelligent Monitoring and Information Technology Research, Hangzhou, China; ^3^Key Laboratory of Forestry Perception Technology and Intelligent Equipment of the State Forestry Administration, Hangzhou, China; ^4^College of Food and Health, Zhejiang Agriculture and Forestry University, Hangzhou, China; ^5^Lin’an District Agricultural and Forestry Technology Extension Centre, Hangzhou, China

**Keywords:** walnut kernels, grading, MDA contents, partitionings, ResNet152V2-SA-SE

## Abstract

Walnut grading is an important step before the product enters the market. However, traditional walnut grading primarily relies on manual assessment of physiological features, which is difficult to implement efficiently. Furthermore, walnut kernel grading is, at present, relatively unsophisticated. Therefore, this study proposes a novel deep-learning model based on a spatial attention mechanism and SE-network structure to grade walnut kernels using machine vision to ensure accuracy and improve assessment efficiency. In this experiment, we found through the literature that both the lightness (L* value) and malondialdehyde (MDA) contens of walnut kernels were correlated with the oxidation phenomenon in walnuts. Subsequently, we clustered four partitionings using the L* values. We then used the MDA values to verify the rationality of these partitionings. Finally, four network models were used for comparison and training: VGG19, EfficientNetB7, ResNet152V2, and spatial attention and spatial enhancement network combined with ResNet152V2 (ResNet152V2-SA-SE). We found that the ResNet152V2-SA-SE model exhibited the best performance, with a maximum test set accuracy of 92.2%. The test set accuracy was improved by 6.2, 63.2, and 74.1% compared with that of ResNet152V2, EfficientNetB7, and VGG19, respectively. Our testing demonstrated that combining spatial attention and spatial enhancement methods improved the recognition of target locations and intrinsic information, while decreasing the attention given to non-target regions. Experiments have demonstrated that combining spatial attention mechanisms with SE networks increases focus on recognizing target locations and intrinsic information, while decreasing focus on non-target regions. Finally, by comparing different learning rates, regularization methods, and batch sizes of the model, we found that the training performance of the model was optimal with a learning rate of 0.001, a batch size of 128, and no regularization methods. In conclusion, this study demonstrated that the ResNet152V2-SA-SE network model was effective in the detection and evaluation of the walnut kernels.

## 1. Introduction

Walnut (*Carya cathayensis* Sarg.) are one important woody nut tree species of the genus *Carya* of Juglandaceae family, mainly distributed in China (1). A popular agricultural commodity, walnuts have a high nutritional content and considerable health advantages (2–5). Walnut processing depends heavily on the quality of the walnuts. At present, the quality identification and grading of walnuts is usually performed manually, which has the following drawbacks: (1) it relies on subjective (and hence unreliable) sensory evaluation, primarily through methods such as peeling and observing the kernel, smelling, and tasting, all of which require professional skill; (2) accurate quality testing requires specialized testing tools; (3) the testing process is complicated and time consuming, making it unfeasible for small-scale production firms at present, and (4) the existing grading is basic and only grades kernels as good or bad. Therefore, to increase the effectiveness and accuracy of grading, an intelligent walnut grading system is needed.

In recent years, machine learning has been used extensively in agriculture (6). In traditional feature-selection algorithms, features are extracted and selected from a feature vector by principal component analysis (PCA) (7). However, PCA tends to disregard information about features with a low contribution ratio. Sometimes, features with lower contribution rates instead contain crucial information. Furthermore, feature selection often requires manual determination of the appropriate features of images, which is subjective and time-consuming. Therefore, many researchers have employed deep-learning models for feature extraction in machine learning-based grading research (8, 9). The researchers used deep-learning models to automatically extract features from various images and enabled the models to focus intelligently and selectively on features with a low contribution ratio (10).

At present, deep-learning models are superior to traditional machine learning models in grading agricultural products (11). In the rice classification study, the deep-learning model enabled quick, accurate, and precise grading, minimizing pre-processing, and eliminating the need for manual feature extraction. The EfficientNet-B0 model achieved scores for class accuracy of 98.33, 96.51, 95.45, 100, 100, 99.26, and 98.72% for healthy, full chalky, chalky discolored, half chalky, broken, discolored, and normal damage classes, respectively (12). At present, pre-training models based on transfer learning are increasingly being utilized by academics, and can minimize the training time for models (13). However, deep-learning models are still deficient, and it is difficult to control the attention of models in the task of image recognition. As a result, network architectures with enhanced network recognition weights are currently emerging (14), and different models and weight assignments will yield various outcomes in agricultural grading models.

In the existing grading of agricultural products, the main criterion of grading is still appearance (15, 16). However, the biochemical properties of the produce also have an impact on the grade of the produce (17). Grading based on appearance alone is not very effective. As a result, genotype research specifically designed for agricultural products was developed (18, 19). These type of research involves many biochemical properties to achieve the grading of agricultural products. Although this type of research can select the best genotypes of produce, each genotype experiment requires the measurement of many biochemical property; therefore this method cannot easily be applied to the grading of agricultural products.

This study utilized the walnut kernel oxidation phenomenon to establish a correlation between the lightness (L* values) of the appearance of the walnut kernel and the MDA content of the kernels. The L* values of the appearance of the walnut kernels was used to define partitionings for grading of walnuts, and the walnuts’ MDA content was used to validate the appropriateness of these partitionings. Subsequently, a deep-learning model was used to train images of walnut kernels, resulting in the intelligent grading of the walnut kernels. The following are the research contributions of this study:

(1) Walnut kernel oxidation phenomena were used to link the L* values of the appearances of the kernels to their MDA content. The L* values were used to create partitionings for clustering the samples, and the MDA content for validation the rationality of these partitionings. It was demonstrated that the L* values could be one of the features of the walnut kernels learned by the deep-learning model. Thus, a correlation between the walnut kernel images and the quality of the walnuts was established. Subsequent studies of walnut kernels could use the level of L* value reflects the degree of oxidation of the walnut kernel, eliminating the need to measure the degree of oxidation of walnut kernels.

(2) To create a ResNet152V2-SA-SE model for the grading study of the walnut kernel appearance images, a residual model based on transfer learning with a spatial attention mechanism and an SE-network structure was used.

(3) By combining deep-learning with an analysis of biochemical properties, a new walnut kernel grading method was defined. It also altered the traditional manual grading method to make grading easier. By matching the appearance features with the biochemical property, future research on agricultural products will only need to measure the appearance features to obtain sufficiently accurate of biochemical properties.

## 2. Materials and methods

The walnuts were harvested in September from a plantation located in Lin’an, Zhejiang, China (30°17′24.2″N; 118°57′1.728″). The walnuts used were uniform in size (diameter: 2–2.5 cm), and harvested from mature trees. The traditional post-harvest processing of walnuts mainly consists of washing and screening, drying, machine hulling, and manual grading. As the main objective of this paper is to change the manual grading method, the walnut kernels after machine hulling were used as the experimental sample.

### 2.1. Experimental plan

**FIGURE 1:**
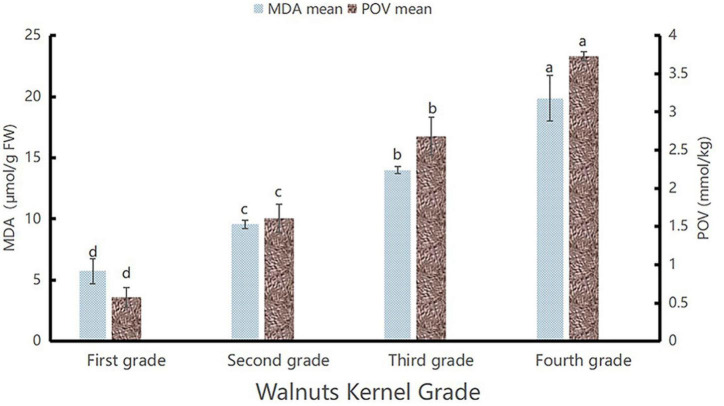
MDA and peroxidation value (POV) diagram. The highest values of POV and MDA contents are marked as “a”, the second highest as “b”, the third highest as “c”, and the lowest as “d”.

In previous studies, researchers have shown that the lightness (L* values) of the appearance of the walnut kernels decreased progressively with prolonged storage durations, accompanied by an increase in the degree of oxidation (20). In addition, it is known that the degree of oxidation of a walnut product correlates with its quality (21). And the MDA is a major product of lipid peroxidation in unsaturated fatty acids, which is considered an accurate and sensitive parameter for assessing oxidative deterioration (22). Therefore, we assume that if we want to correlate the appearance and quality of walnut kernel, the oxidation degree can be used as the intermediate value, and the L* value of appearance can be correlated with the MDA content. To achieve this, we determined to measure the MDA contents and peroxidation value (POV) of the Lin’an walnut kernel samples. The research results showed that the pattern of change in MDA content and POV was similar, as shown in Figure 1. This proved that the MDA content was positively correlated with POV because the level of POV directly reflects the degree of oxidation of walnut kernels, and the higher the POV the higher the degree of oxidation of walnut kernels, so it proved that the MDA content was positively correlated with the degree of oxidation. Because the L* value of walnuts is negatively correlated with the degree of oxidation (20), we conclude that a decline in the L* value of the appearance of the walnut kernels was accompanied by an increase in the POV and MDA content in the walnut kernels, which was consistent with the results of Pei et al. (23).

Finally, this study correlated the L* value of the appearance of walnut kernel with the MDA content through oxidation phenomena.

**FIGURE 2:**
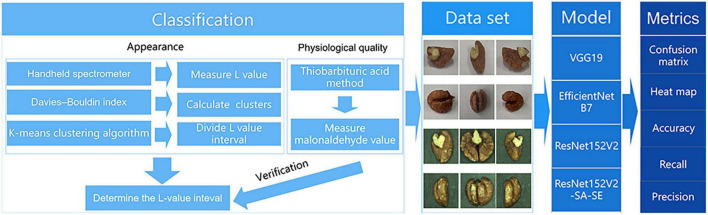
Project technology roadmap.

Figure 2 depicts the technology roadmap for this study.

The first step was building an association model. The L* values of the appearance of the walnut kernels were clustered to establish four partitionings (24). The MDA content of the walnut kernel was utilized to validate these partitionings.

Step two involved the creation of a dataset. For this, we utilized a visual motion platform, using Android and Apple mobile devices to capture photographs of the walnut kernels under different lighting conditions and those with different shapes and textures to exclude fixed features other than L* values. The dataset was built based on four partitions of the L* values of the appearance of the walnut kernels. We consider the four partitions as four categories. Android phones, Apple phones, and visual motion platforms were applied to each partition.

In the third step, the models were trained and compared. Among the EfficientNetB7, VGG19, and ResNet152V2 models, the ResNet152V2 model exhibited the best performance. The ResNet152V2-SA-SE model was constructed by adding a spatial attention mechanism and a squeeze-and-excitation network (SENet) structure to refine the feature map weight assignment of the ResNet152V2 model.

### 2.2. Pre-experimentation

The source of materials for this study was Lin’an District, Hangzhou City, Zhejiang Province, which is the primary walnut-producing region in China (2). We used a CANY XP205 quasi-microbalance, a Kaida TG16G table microcentrifuge, 7G UV-6800A spectrophotometers, and a Konica Minolta CR-10PLUS color reader.

#### 2.2.1. Intrinsic and extrinsic quality correlation modeling

The L* values of the appearance of the walnut kernels were used to construct partitionings, and the MDA content of the walnut kernels was used to validate the rationality of these partitionings.

#### 2.2.2. Appearance inspection

**FIGURE 3:**
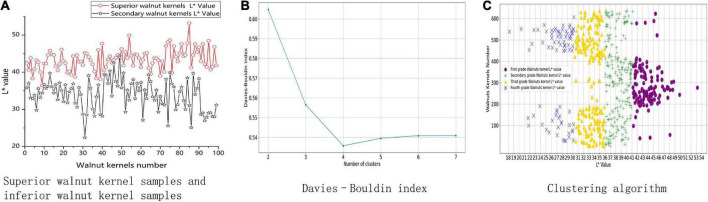
Walnut kernel association model construction process. **(A)** Shows measurements of L* values for superior and inferior walnut kernels. **(B)** Shows the result of the Davies Bouldin index. **(C)** Shows the result of clustering algorithm.

In this experiment, 200 walnut kernels were chosen; half of the walnuts were high quality, and the other half were inferior. The L* values of the appearance of the walnut kernels were measured using a quasi-microbalance. The test showed that the superior walnut kernels generally had higher L* values than inferior walnut kernels. The trials demonstrated that, as shown in Figure 3A, there was a correlation between L* value and quality.

#### 2.2.3. Partitioning clustering

To determine if the L* values of the appearance of the walnut kernels can be used as a walnut kernel grading feature, this study graded the kernels using the L* value of the appearance as the primary index for grading. A total of 636 walnut kernels were chosen at random. The Davies–Bouldin index (DBI) (25) plot is shown in Figure 3B.

The number of clusters (*K* = 4) for the K-means clustering algorithm was determined by DBI (24, 25) and the L* values were divided into four partitionings. As shown in Figure 3C, these partitionings of the L* values were determined to be as follows: (0, 30), (30, 36), (36, 41), and (41, 54). Thus, (0, 30) was the lowest grade, and (41, 54) the highest.

#### 2.2.4. Quality inspection

**FIGURE 4:**
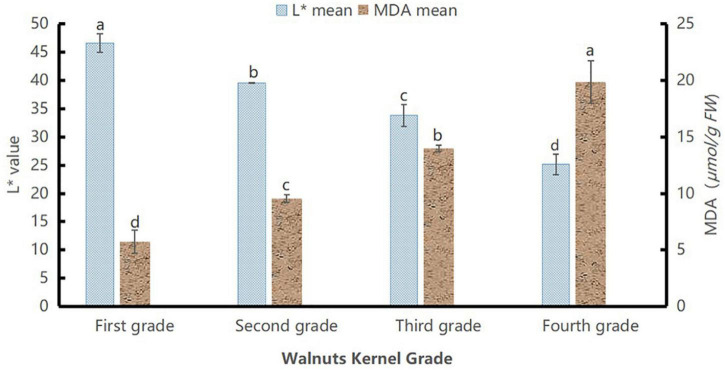
L* values and malondialdehyde content of walnut samples of different grades. Different lowercase letters indicate significant differences between different grades of the same index (*P* < 0.05).

To evaluate the L* values and MDA content levels of the samples separately, we chose a total of 60 walnuts, with 15 of each grade. The results are displayed in Figure 4.

The MDA content of the walnut kernels showed an increasing trend. The MDA content of the kernels increased with the aging degree. Figure 4 shows significant differences among grades as the aging degree increased from grade I to grade IV (*P* < 0.05), whereas the L* values decreased with age, showing significant differences among grades (*P* < 0.05). This indicated that the MDA content and L* values represented the intrinsic quality and extrinsic quality attributes, respectively, fully characterized the differences among different grades, and could fully describe the differences between kernels of different aging degrees. Based on the MDA content and L* values, the walnut kernels were graded as follows: grade I (41, 54); grade II (36, 41); grade III (30, 36), and grade IV (0, 30), shown in Table 1. By experimenting with MDA content, we verified the rationality of L* value partitionings.

**TABLE 1:** Ranges for walnut kernel grades.

Walnuts kernel grade	L* value interval
First grade	L* value is greater than 41 and less than or equal to 54
Second grade	L* value is greater than 36 and less than or equal to 41
Third grade	L* value is greater than 30 and less than or equal to 36
Fourth grade	L* value is greater than 0 and less than or equal to 30

### 2.3. Data acquisition and dataset construction

This study used various image acquisition methods to increase the diversity of the dataset. The study also used deep-learning models for model training so that the models could autonomously learn features other than the L* values of the appearance of the walnut kernels, such as the shape and texture of different grades of the kernels.

#### 2.3.1. Materials and equipment

**FIGURE 5:**
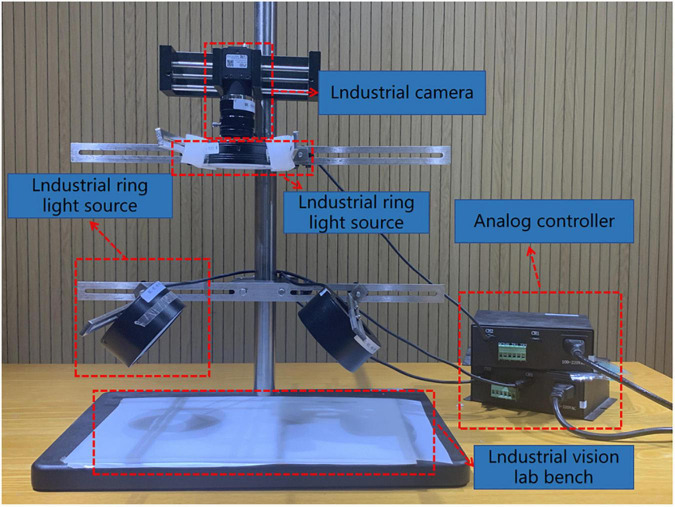
Visual motor platform.

The image acquisition devices used included the iPhone XR mobile phone, Redmi Note 5 mobile phone, and a visual motion platform. Both the Apple XR and Redmi Note 5 smartphones were equipped with 12-megapixel rear cameras. The Apple XR has a larger sensor and an adapted processor that reduces exposure time, and takes lower International Standards Organization values than Redmi Note 5 mobile phone in the same environment, reducing the noise effect. The visual motion platform comprised an ML-SYT-04 industrial vision lab stand, an ML-FA-3517 industrial lens, an ML-RL75-A00-W industrial ring light source, an ML-AS5050-W industrial backlight source, and an MER-132-30G industrial camera. The brand of the visual motion platform was DAHENG IMAGING. The arrangement of the visual motion platform is illustrated in Figure 5.

#### 2.3.2. Data acquisition and data enhancement

**FIGURE 6:**
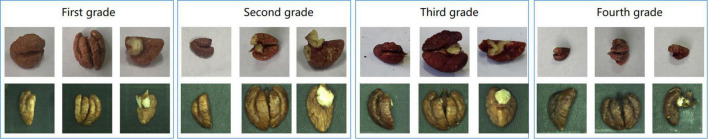
Example dataset.

Each intact walnut kernel was divided into multiple flaps to increase the amount of data. To improve the robustness of the model after training and to eliminate influencing factors other than the L* value, images of walnut kernels were taken under different lighting conditions and with varied shapes and textures. Part of the dataset is shown in Figure 6. A total of 4,395 original images of the four grades were obtained for the experiment, including 947 first-grade walnut kernels, 1,183 second-grade walnut kernels, 1,020 third-grade walnut kernels, and 1,245 fourth-grade walnut kernels. Considering the uneven distribution of the grades of the walnut kernels, firstly, the original dataset was divided into a training set, validation set, and test set at a ratio of 8:1:1, and subsequently, the dataset was expanded using random rotation plus random cropping. The expanded dataset consisted of 1,607 first-grade walnut kernels, 1,548 second-grade walnut kernels, 1,558 third-grade walnut kernels, and 1,500 fourth-grade walnut kernels.

### 2.4. Model construction

#### 2.4.1. Model training parameter settings

The images of the training set were dynamically expanded using the image data generator during image training. After the application of random rotation plus random cropping, the training images were then subjected to the following transforms: random shear transformation, with a parameter value of 0.2; random zoom, with a parameter value of 0.2; and horizontal and vertical shift, with a parameter value of 0.1. This was to train the model thoroughly and increase the generalizability of the training results.

#### 2.4.2. Residual network structure

Deep residual networks are referred to as ResNets (26). Before the emergence of residual networks, the deeper the network layers, the more apparent the gradient disappearance of the model, which results in less accurate models. Considering that the walnut kernel images collected in this experiment were derived from different environments, and noting that a deeper residual model can identify more features, among the pre-trained models, the deepest ResNet152V2 model was chosen in this study.

#### 2.4.3. ResNet152V2-SA-SE network construction

(1) Transfer learning

Transfer learning is a deep-learning method that aims to accelerate a model’s training process (13). Therefore, for this study, we decided to use the ResNet152V2 pre-trained model from the 2015 ImageNet competition for transfer learning.

(2) Spatial attention mechanism

In this study, the use of the spatial attention mechanism (27) in the recognition task enabled the model to focus more on key features such as the shape and texture of the walnut kernels, and thus ignore non-key features such as the background of the kernels. We added a convolutional layer with 2,048 convolutions and a convolutional kernel size of 1 × 1 after the post_relu layer of the ResNet152V2 pre-training model to enhance the extraction of image features when building the model. Subsequently, we linked this layer to the spatial attention mechanism. Finally, a new convolutional layer was formed to refine the weighting of image features. The formula for the generated feature information, M_*s*_, was as follows:


(1) $$M_{S}\,\left(C\right)=\sigma \,\left(f^{7\times 7}\,\left(\left[A\,v\,g\,P\,o\,o\,l\,\left(C\right);M\,a\,x\,P\,o\,o\,l\,\left(C\right)\right]\right)\right)$$

where σ denotes the hard sigmoid activation function, f ^7 × 7^ denotes a convolutional neural network structure with 7 × 7 convolutional kernels, and AvgPool(C) and MaxPool(C) are the global average pooling and maximum global pooling, respectively of the input image C. Subsequently, the output walnut kernel feature map was fed into the Batch Normalization (BN) layer to speed up the training and convergence of the network and prevent the gradient disappearance problem. Finally, the walnut kernel feature map from the BN layer was output and denoted X after being multiplied by the original map and passed into the next layer.

(3) SENet

SENet was divided into the squeeze operation and the excitation operation (28). The squeeze operation extracts each feature map as scalar data using a global pooling operation. The key formula is as follows.


(2) $$k_{n}=F_{s\,q}\,\left(u_{n}\right)=\frac{1}{H\times W}\,\sum_{x=1}^{H}\sum_{y=1}^{W}u_{n}\,\left(x,y\right)$$

In Eq. 2 the sum of all pixel values of the H × W walnut kernel feature map is calculated and divided by H × W to obtain Fsq. The squeeze operation obtains the global description of each walnut kernel feature map, which opens the connection between the channels. The excitation operation is used to obtain the relationship between the channels and is expressed as follows:


(3) $$p=F_{e\,x}\,\left(k,W\right)=\sigma \,\left(g\,\left(k,W\right)\right)=\sigma \,\left(W_{2}\,\delta \,\left(W_{1}\,k\right)\right)$$

Equation 3 describes the two full-connection operations and the sigmoid activation function to obtain the corresponding channel weights. σ is the ReLU function, and W_1_ is the first full-connection operation for dimensionality reduction. W_2_ is the parameter of the second full-connection operation to restore the correct dimensionality to the input dimension. After two full joins, the weight normalization operation is performed on each walnut kernel feature map using the sigmoid activation function.

After the weight p is obtained, it is multiplied by the original walnut kernel feature map to obtain the walnut kernel feature map with weights, as shown in Eq. 4.


(4) $$\tilde{X}=F_{\text{scale}}\,\left(u_{n},p_{n}\right)=p_{n}\cdot u_{n}$$

The spatial attention mechanism enhances the acquisition of spatial information about the walnut kernel in the image. Then the SENet applies the weights extracted by the channels to the feature maps so that the channels are adaptively weighted, thus allowing the feature maps with a large role to have an increased impact on the results.

**FIGURE 7:**
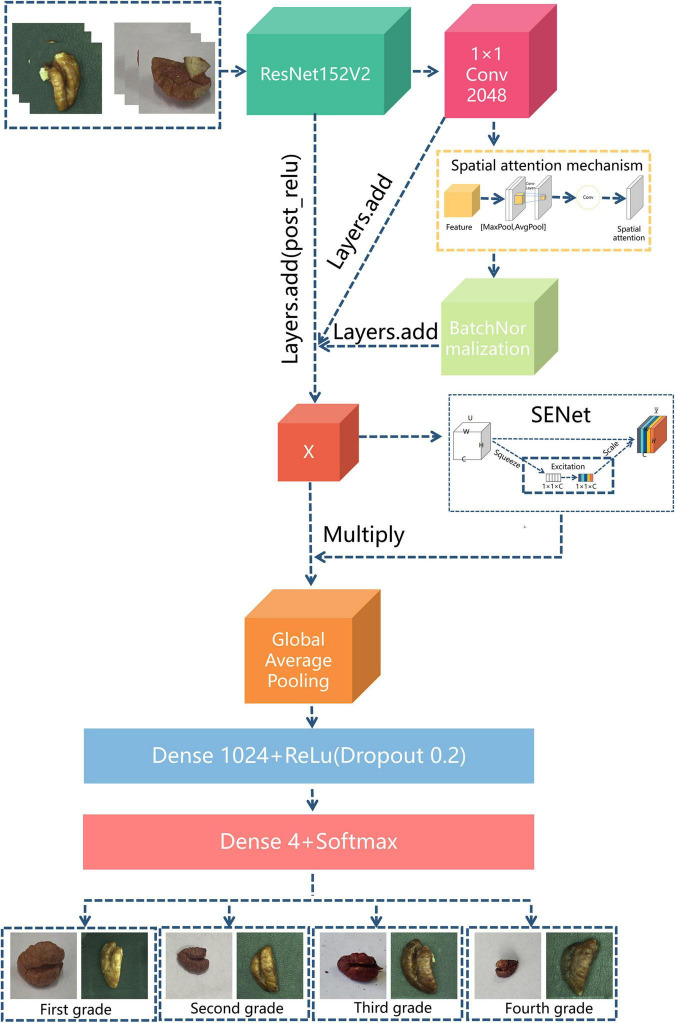
ResNet152V2-SA-SE network structure.

The overall network structure of the ResNet152V2-SA-SE model is shown in Figure 7.

## 3. Results and analysis

### 3.1. Evaluation indicators

In this study, precision, recall, and accuracy were used as validation metrics to evaluate the model.


(5) $$A\,c\,c\,u\,r\,a\,c\,y=\frac{T\,P+T\,N}{T\,P+T\,N+F\,P+F\,N}$$

(6) $$R\,e\,c\,a\,l\,l=\frac{T\,P}{T\,P+F\,N}$$

(7) $$P\,r\,e\,c\,i\,s\,i\,o\,n=\frac{T\,P}{T\,P+F\,P}$$

where TP denotes a true positive, TN a true negative, FP a false positive, and FN a false negative.

### 3.2. Experimental environment

In this study, experiments were carried out on Windows 10 using the Keras framework under the TensorFlow framework. The experiments were conducted using an Intel(R) Core(TM) i9-10920X processor running at 3.50 GHz, 64.0 GB of memory, and an Nvidia GeForce RTX 3080 graphics card. The software was Python 3.6.0 and TensorFlow 2.4.1. Every model used transfer learning to accelerate model training. A convolutional layer of 1,024 neurons was added to reduce the impact of feature acquisition on grading results. During model training, the learning rate was set to 0.001, the batch size to 128, and the epoch to 100.

### 3.3. Model training and test results

#### 3.3.1. Performance assessment for VGG19, EfficientNetB7, and ResNet152V2

(1) Model comparison

The EfficientNetB7 model used in this experiment contained many parameters and a large model size, as shown in Table 2.

**TABLE 2:** Comparison of the three models.

Model name	Number of parameters	Model size (MB)	Average model training time (s)
VGG19	20,553,796	78.4	96
EfficientNetB7	66,724,251	256	103
ResNet152V2	*60,433,924*	231	104

This is because the EfficientNetB7 model is enhanced in three dimensions (depth, width, and resolution), increasing its structural complexity. The VGG19 model had the least number of parameters, the smallest model size, and the quickest training time, but its structure was simpler, and gradient disappearance was more likely to be caused by a high number of picture features. Although the ResNet152V2 model had a deeper model layer, the number of parameters and model size of ResNet152V2 were still smaller than the EfficientNetB7 model.

(2) Comparison of test results

The test results of the three models are shown in Table 3.

**TABLE 3:** Test results of the three models.

Model name	Recognition precision of each level/%				Recognition recall of each level/%			
	First grade	Second grade	Third grade	Fourth grade	First grade	Second grade	Third grade	Fourth grade
VGG19	0.0	0.0	27.0	6.7	0.0	0.0	60.8	12.1
EfficientNetB7	22.5	30.2	25.7	51.5	30.2	43.8	19.0	22.8
ResNet152V2	97.4	67.9	86.9	98.5	95.0	86.9	73.9	87.9

According to the analysis of the models’ precision rates, the VGG19 model could not distinguish between first- and second-grade walnut kernels. The third-grade walnut kernel recognition precision for the VGG19 model was very low, at just 27%. The average precision rate of the EfficientNetB7 model was only 32.5%. Only a very small proportion of the walnut kernel images could be accurately identified in the detected walnut kernel images because the average precision of the two types of models was extremely low. The ResNet152V2 model, in contrast, had an average precision of 87.7%, which was significantly greater than that of the models mentioned above.

The recall rate of the model was analyzed. Although the recall rate of VGG19 for the third-grade walnut kernel images reached 60.8%, the recognition precision of VGG19 for the third-grade walnut kernel images was only 27.0%, indicating that the VGG19 model had a low efficiency due to the misrecognition of other grade walnut kernels as third-grade walnut kernels. The average recall rate of the EfficientNetB7 model was only 29%. The average recall rates of both models were extremely low; furthermore, the percentage of correctly identified walnut kernel images was too low to be used in the real walnut kernel prediction stage. In contrast, the average recall of the ResNet152V2 model reached 85.9%, and its performance was much better than that of the models mentioned above.

In conclusion, the VGG19 model and the EfficientNetB7 model both exhibited poor performance. The average precision rate of the ResNet152V2 model was 87.7%, higher than the 79.3 and 55.2% of the VGG19 and EfficientNetB7 models, respectively. The average recall rate of the ResNet152V2 model was 85.9%, higher than the 67.7 and 56.9% of the VGG19 model and the EfficientNetB7 model, respectively.

(3) Analysis of the reasons for differentiation

**FIGURE 8:**
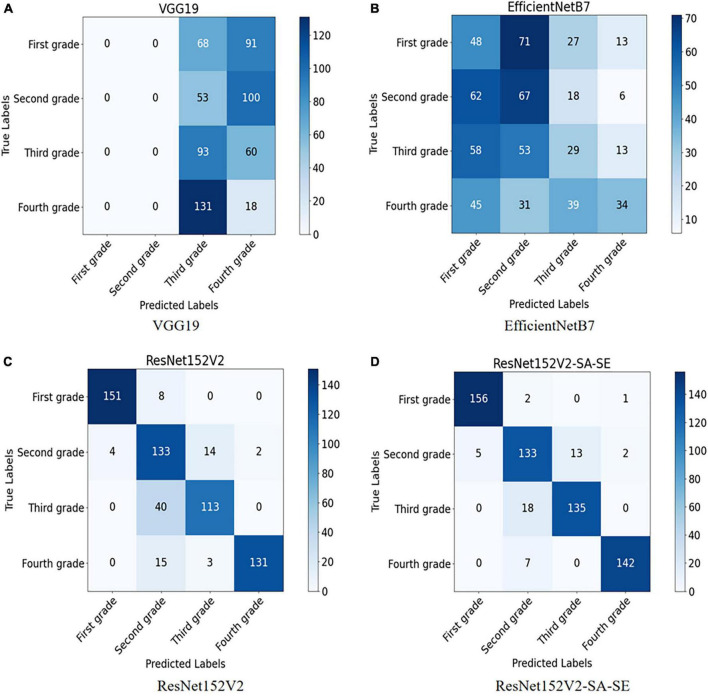
**(A)** Confusion matrix model of VGG19 model; **(B)** confusion matrix model of EfficientNetB7 model; **(C)** confusion matrix model of ResNet152V2 model; and **(D)** confusion matrix model of ResNet152V2-SA-SE model.

**FIGURE 9:**
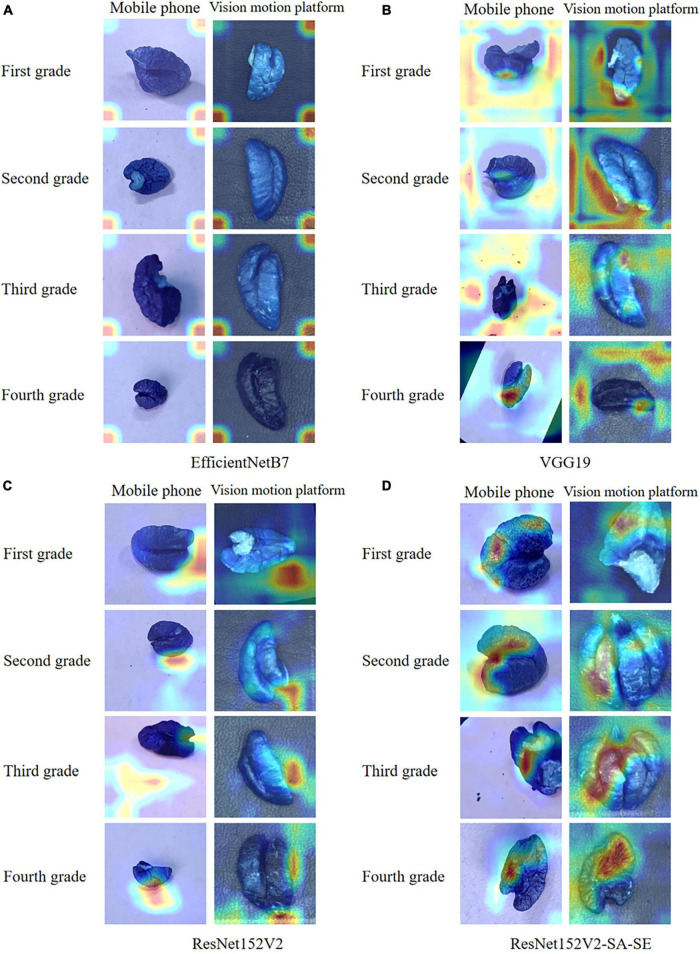
**(A)** Heat map of the EfficientNetB7 model; **(B)** heat map of the VGG19 model; **(C)** heat map of ResNet152V2; and **(D)** heat map of ResNet152V2-SA-SE.

To further explore the test set identification error problem of the VGG19 and EfficientNetB7 models, the confusion matrix and the heatmap of the above model were drawn. The confusion matrix is shown in Figures 8A, B, and the heatmaps are shown in Figures 9A, B.

As seen in the confusion matrix for the test results of the VGG19 network model, the recognition accuracy of the network for predicting the four walnut kernel grades was relatively low, with 0% correct recognition of both first- and second-grade walnut kernel images and 6.7% correct recognition of fourth-grade walnut kernel images. This is because, when learning the image features of walnut kernels, as a result of the deeper layers of the VGG19 model network, when updating the network weights by back-propagation of the gradient, the gradient will propagate exponentially and thus approach 0. Eventually, the VGG19 model experiences gradient disappearance and cannot learn which image features are useful for recognition.

For the EfficientNetB7 network model, the confusion matrix indicated that the rate of recognition of the model was 22.5 and 25.7% for first- and third-grade walnut kernels, respectively, which was still a low rate of recognition. However, these rates were slightly better than those of the VGG19 network model. Observing the heatmap, we found that the EfficientNetB7 model focused its observations on the edges of the walnut kernel images, which was the main reason for the poor accuracy. Because walnut kernels, as smaller recognition targets, have less obvious physical features such as texture and shape, the model needed to analyze the walnut kernel images for deeper feature information during recognition and thus notice these small differences. For this reason, the depth of the model was a major influence on the grading recognition of walnut kernel images. As EfficientNetB7 balances the other two dimensions by reducing the weights assigned to the depth of the model. The weights assigned to the depth of the model were reduced weakening the expressiveness of the model, which meant that the model was unable to extract subtle features between the images of walnut kernel and ended up only locating important features of the image roughly at the edges of the image.

Based on the test results of the ResNet152V2 network model, the problem of disappearing gradients in the VGG19 network model was mitigated by adding the residual structure. Compared with the EfficientNetB7 model, the ResNet152V2 model was able to refine the subtle feature information between the walnut kernel images adequately with a deeper model depth. Therefore, the ResNet152V2 model outperformed the VGG19 and EfficientNetB7 models in terms of both model precision and recall. This study improved and optimized the ResNet152V2 model.

#### 3.3.2. Performance assessment for ResNet152V2 model versus ResNet152V2-SA-SE model

(1) Model comparison

A spatial attention mechanism and an SE-network structure were introduced to ResNet152V2. The spatial attention mechanism enhanced the acquisition of spatial information about the walnut kernel in the image. The SE structure gave adaptive weights to channels so that feature maps with large roles had more impact. The new model was referred to as ResNet152V2-SA-SE. Although the ResNet152V2-SA-SE model increased the number of parameters and model size compared to ResNet152V2, the new model had a reduced training time. We compared ResNet152V2-SA-SE to ResNet152V2, and the results are displayed in Table 4.

**TABLE 4:** Comparison of the two models.

Model name	Number of parameters	Model size (MB)	Average model training time (s)
ResNet152V2	*60,433,924*	231	104
ResNet152V2-SA-SE	*65,165,030*	249	102

(2) Comparison of test results

The test results of the two models are shown in Table 5. The recognition precision of the ResNet152V2-SA-SE model was 15.2 and 4.3% higher than that of the ResNet152V2 model for second- and third-grade walnut kernels, respectively. Compared with the ResNet152V2 model, the ResNet152V2-SA-SE model recognized more walnut kernel grade images correctly.

**TABLE 5:** Test results of the SA-ResNet152V2 model and ResNet152V2-SA-SE model.

Model name	Recognition precision of each level (%)				Recognition recall of each level (%)			
	First grade	Second grade	Third grade	Fourth grade	First grade	Second grade	Third grade	Fourth grade
ResNet152V2	97.4	67.9	86.9	98.5	95.0	86.9	73.9	87.9
ResNet152V2-SA-SE	96.9	83.1	91.2	97.9	98.1	86.9	88.2	95.3

The recognition recall of the ResNet152V2-SA-SE model for first-grade, third-grade, and fourth-grade walnut kernels were 3.1, 14.3, and 7.4% higher, respectively than those of the ResNet152V2 model. These results indicated that the ResNet152V2-SA-SE model correctly identified first-grade, third-grade, and fourth-grade walnut kernel images at a higher rate.

In summary, the average precision and recall of the ResNet152V2-SA-SE model were higher than those of the ResNet152V2 model by 4.6 and 6.2%, respectively. Thus, we concluded that the ResNet152V2-SA-SE model performed better than the ResNet152V2 model in the test set overall. Compared with the ResNet152V2 model, the ResNet152V2-SA-SE model had a higher recognition accuracy.

(3) Analysis of the reasons for the performance differences

To further explore the advantages and disadvantages of the ResNet152V2 model and the ResNet152V2-SA-SE model, the confusion matrices of the two models were generated, as shown in Figures 8C, D. The walnut kernel heatmaps obtained for the ResNet152V2 model and ResNet152V2-SA-SE model are shown in Figures 9C, D.

The confusion matrices demonstrated that, compared to ResNet152V2, the ResNet152V2-SA-SE model recognized 3.1, 14.4, and 7.4% more first-, third-, and fourth-grade walnut kernels, respectively.

According to the heatmap, the ResNet152V2-SA-SE model, which was based on the ResNet152V2 model, focused on obtaining the location and intrinsic details of the walnut kernel while paying less attention to unimportant details. In particular, it was clear from the images obtained from the visual motion platform that the ResNet152V2-SA-SE model concentrated on crucial information that was no longer restricted to the walnut kernel itself. It also focused on information regarding the walnut kernel’s location and intrinsic image information. The detection of incomplete walnut kernel images was made easier, and the model generalizability was greatly increased by focusing on location information.

In summary, the spatial attention mechanism enhanced the acquisition of spatial information about the walnut kernel in the image, and the SENet applied the weights extracted by the channels to the feature maps so that the channels were adaptively weighted, thus allowing the feature maps with a large role to have an increased impact on the results. This operation enabled the model to focus more on the recognition subject common to all image predictions.

#### 3.3.3. Overall comparison of the four types of models

Two measures, validation set accuracy and test set accuracy, were introduced in this study to examine the overall differences between the four models, as shown in Table 6.

**TABLE 6:** Test results of the four types of models.

Model name	Average accuracy of validation set/%	Average precision of test set/%	Average recall of test set/%	Average accuracy of test set/%
VGG19	88.5	8.4	18.2	18.1
EfficientNetB7	25.7	32.5	29.0	29.0
ResNet152V2	91.6	87.7	85.9	86.0
ResNet152V2-SA-SE	91.8	92.3	92.1	92.2

Table 6 show that the average accuracy of the validation set of the ResNet152V2-SA-SE model was 0.2, 66.1, and 3.3% higher than that of the ResNet152V2, EfficientNetB7, and VGG19 models, respectively. The average precision of the test set was improved by 4.6, 59.8, and 83.9%, respectively, and the average recall of the test set was improved by 6.2, 63.1, and 73.9%, respectively. The accuracy of the test set was improved by 6.2, 63.2, and 74.1%, respectively.

In summary, in the model training phase, the ResNet152V2-SA-SE and ResNet152V2 models demonstrated advantages over the VGG19 and EfficientNetB7 model. In the model testing phase, the ResNet152V2-SA-SE model exhibited outstanding performance, by accounting for the global situation, grasping the key points, and ignoring the non-critical information. Based on the experiments, the ResNet152V2-SA-SE model exhibited high accuracy in walnut kernel grading.

## 4. Influence of each factor on model training

The ResNet152V2-SA-SE model was used in the experiments to assess the effects of three elements on the model during training: learning rate, regularization, and batch size. Epochs were set to 100 to examine the effects of various factors on model training.

### 4.1. Effect of learning rate on model training

**FIGURE 10:**
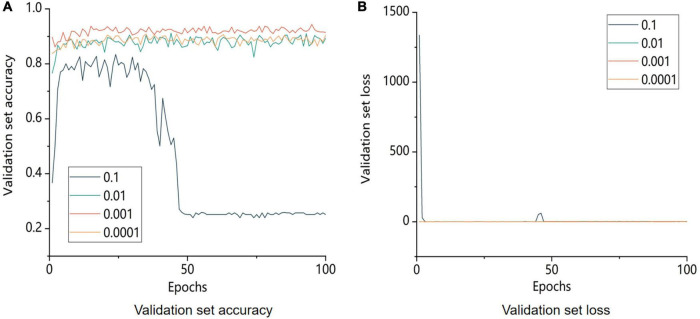
Comparison of accuracy and model loss of the validation set with different learning rates. 0.1, 0.01, 0.001, and 0.0001 are the learning rate parameters set by the model, respectively. **A** is the image of the change in accuracy of the validation set and **B** is the image of the change in loss value of the validation.

The learning rates were set to 0.1, 0.01, 0.001, and 0.0001 to test the training results with various learning rates. The training results are displayed in Figure 10.

By comparing the accuracy of the validation set with the loss for various learning rates, it can be seen that the size of the learning rate was a significant influencing factor for the trained model. The identification accuracy for the validation set gradually improved as the learning rate gradually decreased. However, when the model’s learning rate approached 0.001, the validation set accuracy of the model reached its maximum. Similar outcomes were obtained with further learning rate reduction compared to the model learning rate of 0.01. This finding demonstrated that when the learning rate was too high, it was difficult for the model to converge, whereas when the learning rate was too low, it was impossible for the model to capture the important parts of an image during training. In conclusion, a learning rate of 0.001 was optimal for model training.

### 4.2. Effect of regularization on model training

**FIGURE 11:**
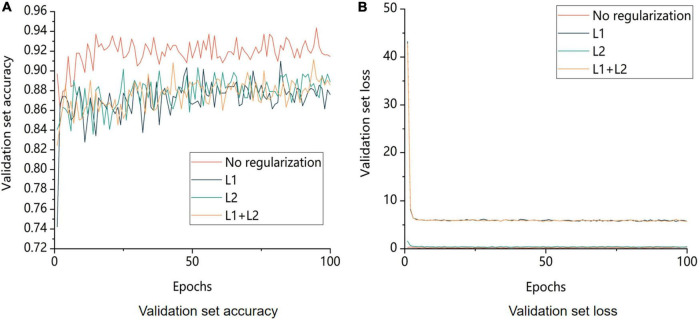
Comparison of validation set accuracy and model loss for different regularization strategies. No regularization is not regularization, L1 is using L1 regularization, L2 is using L2 regularization, L1+L2 is both L1 and L2 regularization being used. **A** is the image of the change in accuracy of the validation set and **B** is the image of the change in loss value of the validation.

Using the control variable approach, the regularization coefficient was set to 0.01, and the results of several regularization strategies were checked. The experimental outcomes are displayed in Figure 11.

The regularization strategy’s main goal was to decrease the possibility of overfitting the model. Figure 11 displays the model’s training outcomes with and without regularization. The model without regularization had greater overall accuracy than the model with regularization. Regularization lowered the likelihood of model overfitting by restricting the model’s capacity to learn; however, these restrictions resulted in the loss of some image features during learning and an increase in bias. In conclusion, adding regularization to the model to increase its accuracy during training was not the best approach in this study.

### 4.3. Effect of different batch sizes on model training

**FIGURE 12:**
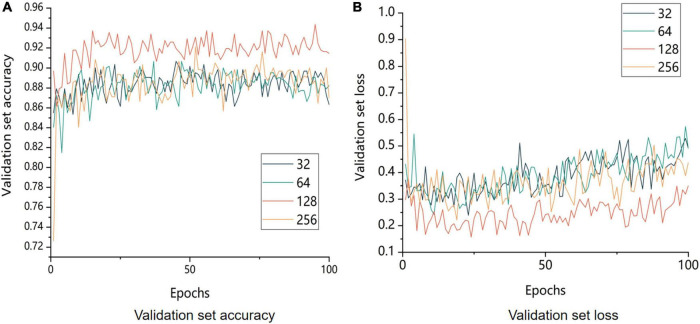
Comparison of accuracy and model loss of the validation set with different batch sizes. 32 means that the batch size is set to 32; 64 means that the batch size is set to 64; 128 means that the batch size is set to 128; and 256 means that the batch size is set to 256. **(A)** Image of the change in accuracy of the validation set and **(B)** image of the change in loss value of the validation.

To verify the results of different batches using the control variable method, the batches were set to 32, 64, 128, and 256. The experimental results are shown in Figure 12.

The size of the model batch was a crucial factor in improving the model performance. The model with a batch size of 128 outperformed other batch sizes in terms of both validation set accuracy and validation set loss, as shown in Figure 12, which displays the comparison of the accuracy of validation set recognition with different batch sizes. On the one hand, a small batch size generally resulted in too much variation between neighboring mini-batches, which negatively affected model convergence. On the other hand, a large batch size decreased the model’s generalization ability.

In conclusion, 128 was the optimal batch size for this experiment.

## 5. Conclusion

(1) This study used oxidation phenomena of walnut kernels to link L* values of the appearance of the walnut kernel and MDA content of the walnut kernel, and then demonstrated experimentally that the L* values of the appearance of the walnut kernel could be used as one of the features for walnut kernel grading. Based on these findings, subsequent researchers can conduct more refined grading experiments and studies on walnut kernel using L* values.

(2) This study enriched the image grading research on walnut kernels by capturing images of walnut kernels using a visual motion platform as well as cell phones, homogenizing the images by rotating and cropping, subsequently expanding the images online using ImageDataGenerator, and using the Keras framework for deep learning. Using the confusion matrix and heat map, we compared each of the three models and selected the optimal ResNet152V2 model for further improvement, and finally, we constructed the ResNet152V2-SA-SE model consisting of the spatial attention mechanism, the SENet, and the ResNet152V2 model. We demonstrated experimentally that the average accuracy of the validation set of the ResNet152V2-SA-SE model was 0.2, 66.1, and 3.3% higher than that of the ResNet152V2, EfficientNetB7, and VGG19 models, respectively; the average precision of the test set was improved by 4.6, 59.8, and 83.9%, respectively; the average recall of the test set was enhanced by 6.2, 63.1, and 73.9%, respectively; and the accuracy of the test set was improved by 6.2, 63.2, and 74.1%, respectively.

(3) The average accuracy of the validation set of the ResNet152V2-SA-SE model reached its maximum with a learning rate of 0.001; after adding various regularization strategies, we discovered that the overall accuracy of the validation set of the model declined with the addition of regularization. As the batch size increased exponentially, the average accuracy of the validation set of the model gradually increased. When the sample size was 128 batches, the validation set accuracy of the model was the highest, the loss lowest, and the model optimal.

The walnut kernel grade recognition problem was successfully solved by the ResNet152V2-SA-SE model in this study, and the challenge of the intelligent grading of small objects with high similarity was addressed as well. At the same time, this study proposed a new grading standard for walnut kernels. The research idea of this study can also inspire subsequent research on grading of various agricultural products, for example, by combining a certain feature of the appearance of the agricultural products with the intrinsic quality of the agricultural products. The initial grading of agricultural products would then be based on their most salient visual features, and eventually, the deep-learning model would learn other features on its own, thus achieving fast and accurate grading of agricultural products. By matching the appearance features with the biochemical property, future research on agricultural products will only need to measure the appearance features to the biochemical property, eliminating the need for repeated measurement. Future research could refine this concept and achieve a more detailed grading. In the future, a hyperspectral camera along with an RGB camera in various artificial and natural light conditions could be used for more in-depth study on the recognition of walnut kernel images.

## Data availability statement

The raw data supporting the conclusions of this article will be made available by the authors, without undue reservation.

## Author contributions

DD, SC, and JZ: conceptualization and methodology. SC and DD: software and validation. SC, HK, and DD: formal analysis and investigation. SC: writing—original draft preparation. SC, DW, and DD: writing—review and editing. SC, XZ, XG, JM, ZL, and DD: funding acquisition. All authors have read and agreed to the published version of the manuscript.
